# Nanostructured Lipid-Based Delivery Systems as a Strategy to Increase Functionality of Bioactive Compounds

**DOI:** 10.3390/foods9030325

**Published:** 2020-03-11

**Authors:** Ariadna Gasa-Falcon, Isabel Odriozola-Serrano, Gemma Oms-Oliu, Olga Martín-Belloso

**Affiliations:** Department of Food Technology, University of Lleida-Agrotecnio Center, Av. Rovira Roure 191, 25198 Lleida, Spain; ariadna.gasa@udl.cat (A.G.-F.); iodriozola@udl.cat (I.O.-S.); goms@udl.cat (G.O.-O.)

**Keywords:** nanoemulsions, multi-layer emulsions, liposomes, bioactive compounds, stability, lipid digestibility, bioaccessibility, absorption, functionality

## Abstract

Acquisition of a healthy lifestyle through diet has driven the food manufacturing industry to produce new food products with high nutritional quality. In this sense, consumption of bioactive compounds has been associated with a decreased risk of suffering chronic diseases. Nonetheless, due to their low solubility in aqueous matrices, high instability in food products during processing and preparation as well as poor bioavailability, the use of such compounds is sometimes limited. Recent advancements in encapsulation and protection of bioactive compounds has opened new possibilities for the development of novel food products. In this direction, the present review is attempting to describe encapsulation achievements, with special attention to nanostructured lipid-based delivery systems, i.e., nanoemulsions, multi-layer emulsions and liposomes. Functionality of bioactive compounds is directly associated with their bioavailability, which in turn is governed by several complex processes, including the passage through the gastrointestinal tract and transport to epithelial cells. Therefore, an overview of recent research on the properties of these nanostructured lipid-based delivery systems with a strong impact on the functionality of bioactive compounds will be also provided. Nanostructured lipid-based delivery systems might be used as a potential option to enhance the solubility, stability, absorption and, ultimately, functionality of bioactive compounds. Several studies have been performed in this line, modifying the composition of the nanostructures, such as the lipid-type or surfactants. Overall, influencing factors and strategies to improve the efficacy of encapsulated bioactive compounds within nanostructures have been successfully identified. This knowledge can be used to design effective targeted nanostructured lipid-based delivery systems for bioactive compounds. However, there is still a lack of information on food interactions, toxicity and long-term consumption of such nanostructures.

## 1. Introduction

The World Health Organization (WHO) reported that by 2020, chronic diseases will be responsible for nearly three-quarters of all deaths worldwide, including ischaemic heart disease, deaths due to stroke and diabetes [1]. The fact that eating habits are relevant in either the appearance or the prevention of these chronic illnesses provides food for thought. People’s awareness of nutrition habits is increasing and has derived into a growing demand for foodstuffs with specific functions for overall good health and well-being.

Not being synthesized by the human body, bioactive compounds need to be incorporated into our daily diet since they are involved in several biological processes, such as the regulation of gene expression in cell proliferation and apoptosis, as well as intermediation in hormone metabolism [2]. In addition, they exhibit antioxidant and anti-inflammatory activities, as well as antibacterial properties, which have drawn the interest of the scientific community studying these compounds [3].

In an attempt to satisfy the consumer’s requests, the food industry is trying to use and incorporate these bioactive compounds within foodstuffs [4]. Nonetheless, they present several limitations, including low water solubility, stability and bioavailability, which can compromise successful incorporation and functionality. The common interest of both consumers and the food industry for healthier products has been translated into an in-depth search of tools in order to overcome these limitations [5]. Researchers have been exploring the possibilities for incorporating bioactive compounds into food and beverages as well as for strengthening their positive health benefits. In this sense, nanotechnology has been envisaged as a potential approach that offers several applications in the food field, such as animal feed, novel foods and food additives. Nanotechnology has been established as a key enabling technology (KET) by the European Commission because it facilitates forward-looking possibilities for the development of food innovative products [6]. Specifically, nanostructured delivery systems consist of tiny droplets within the nanometric range, with the proven ability to carry and protect bioactive compounds. So far, different types of carrier systems have been developed, including nanoemulsions [7], double emulsions [8], multi-layer emulsions [9], liposomes [10] and solid lipid nanoparticles [11], among others. These systems allow the solubilisation, encapsulation and protection of bioactive compounds, but also improve their stability under certain environments such as heat, extreme pH and gastrointestinal fluids [12]. Thus, delivery of bioactive compounds through the gastrointestinal tract is guaranteed and, consequently, their bioavailability and functionality are enhanced. The outstanding properties concerning these systems facilitate the incorporation of bioactive compounds to foodstuffs, thereby conferring an extra nutritional value.

This review will be focused on discussing the use of nanostructured lipid-based delivery systems for enhancing the functionality of lipophilic bioactive compounds.

## 2. Lipophilic Bioactive Compounds

In the last decade, there has been a significant growing interest toward the development of nutraceuticals, which are foodstuffs that provide health and medical benefits [13,14]. Therefore, it is very common in the food and beverage industry to extract bioactive compounds molecules from its original sources and purify them for several applications, including dietary supplements or food fortification [15]. Bioactive compounds are non-essential for human life, but they have been categorised as disease-prevention ingredients due to their antioxidant, anti-tumour, anti-mutagenic, anti-proliferative and anti-inflammatory properties. Numerous studies have proven these health benefits both in vitro (tissue culture studies) [16,17] and in vivo (animal and human intervention trials) [18,19]. These bioactive compounds are mainly found in fruits and vegetables, but also in legumes, nuts, herbs and spices, among others. According to some studies, the European population does not meet the daily recommended intake of fruits and vegetables, which has been set in 400 g/day [20,21]. In addition, special attention has been paid to lipophilic bioactive compounds (carotenoids, flavonoids and vitamins E, D and K), since their incorporation to aqueous-based food products is limited. Furthermore, these compounds are light, temperature and/or oxygen sensible, which can negatively affect their efficacy as health-related agents. Either manufacturing or storage of foodstuffs as well as the extreme conditions found through the digestive tract can lead to degradations of these lipophilic compounds [22]. For instance, high temperature during food processing or acidic environments during the stomach phase can lead to the transformation of β-carotene into oxidation products, such as carotenoid carbocation, which is indicative of compound degradation [23]. Besides that, digestive fluids are also aqueous, which can affect their transport throughout the gastrointestinal tract and the transfer to mixed micelles for further absorption. Thus, a combination of all these factors might imply the loss of lipophilic bioactive compounds and, consequently, compromise the intestine reach, absorption and postprandial delivery to target organs, meaning poor functionality. Nanostructured lipid-based delivery systems might help address these problems concerning bioactive compounds.

## 3. Nanostructured Lipid-Based Delivery Systems

**Figure 1:**
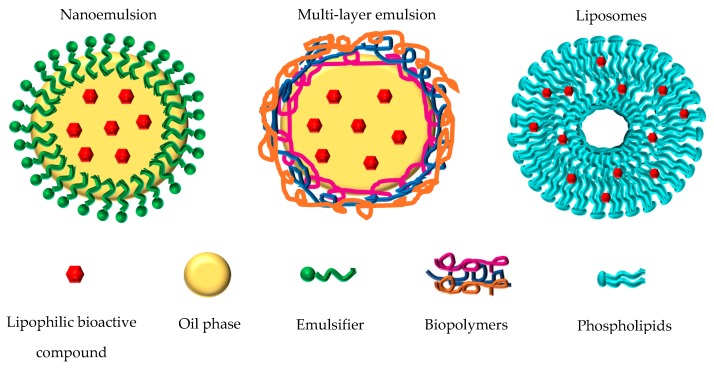
Nanostructured lipid-based delivery systems for encapsulating lipophilic bioactive compounds.

Research studies focused on nanostructured lipid-based delivery systems as a strategy to protect lipophilic bioactive compounds have extensively increased. A brief description of the main nanostructured lipid-based delivery systems for encapsulating such compounds, including nanoemulsions, multi-layer emulsions and liposomes (Figure 1), is outlined below.

### 3.1. Nanoemulsions

Nanoemulsions are oil-in-water emulsions containing nanometric-size oil droplets ranging from 50 to 500 nm [24]. The main components for nanoemulsions formation are oil (lipid phase) and water (aqueous phase). On one hand, lipophilic bioactive compounds are solubilized within the oil before the formation of nanoemulsions. Different oils haven been employed, including corn oil [25], essential oils [26] and medium-chain triglycerides (MCT) (Miglyol^®^ 810 or Miglyol^®^ 812) [7]. On the other hand, there are different components that can be incorporated within the aqueous phase, such as emulsifiers (Tweens, phospholipids, proteins), texture modifiers (sodium alginate, pectin, carrageenan), preservatives and/or antioxidants (parabens, tocopherols), among others. Both the lipid and aqueous phases are immiscible, so they need to be combined in order to disperse the lipid phase (oil droplets) within the aqueous phase (water surrounding oil droplets). For this, the presence of emulsifiers is important since they are surface-active compounds that are adsorbed at the oil–water interface of the droplets, thereby reducing the interfacial tension between both phases and assisting in the stabilisation of the system [27]. Their ability to adsorb at the oil–water interface is also subjected to the molecular structure of each emulsifier [28]. Finally, homogenization of both phases with all the components is an important process in order to form nanoemulsions. For this process, the use of high-energy (high-pressure homogenization, sonication) or low-energy methods (self-emulsification, phase inversion) is required [29]. High energy methods are widely employed for producing these systems, which consist of applying mechanical forces (shear, disruptive) in order to create small oil droplets through their break down [30]. Alternatively, low-energy methods consist of spontaneous emulsification by using the internal chemical energy of the system [31].

The final properties of nanoemulsions will be determined not only by the nature and characteristics of each component forming part of the system [25,32,33] but also by the fabrication employed for producing nanoemulsions [34].

### 3.2. Multi-Layer Emulsions

Multi-layer emulsions are oil-in-water emulsions with oil droplets covered by at least two layers of biopolymers that are electrostatically charged. Several stabilisers, including surfactants (lecithin, sodium dodecyl sulphate, Tween 20) and biopolymers (sodium alginate, pectin, chitosan, β-lactoglobulin) have been extensively used to produce multi-layer emulsions with different lipophilic bioactive compounds, such as lutein [35], resveratrol [9], curcumin [36] and β-carotene [37].

In general, a single-layer emulsion is firstly done, thereby homogenizing the lipid phase (oil containing lipophilic bioactive compounds) and the aqueous phase (charged surfactant or biopolymer). Then the layer-by-layer (LbL) method is used to produce several multilayers by placing biopolymers oppositely charged around the oil droplets.

It should be stated that fabrication of these emulsions is a delicate procedure as instability processes (e.g., bridging flocculation or depletion flocculation) occurs when there is either an excess or lack of polyelectrolytes [38]. In this sense, there are different strategies that can be employed to produce stable multi-layer emulsions: (1) the saturation method, to empirically determine (ζ-potential and/or particle size) the concentration of the biopolymers needed to cover the oil droplets; (2) the centrifugation method, to remove the excess non-adsorbed biopolymers by centrifuging; or (3) the filtration method, whereby the excess of non-adsorbed biopolymers is removed by membrane filtration.

When a suitable concentration of each polyelectrolyte is employed to form multi-layer emulsions, there are two types of stabilisation mechanisms that contribute to the equilibrium of the whole system. On one hand, steric stabilisation occurs when oil droplets are coated with biopolymers that provide a layer around them, which is thick enough to overcome attractive forces between droplets. On the other hand, charges of the stabilisers covering the oil droplets contribute to the electrostatic stabilisation, which avoids that the oil droplets come close to each other [39]. Apart from biopolymer concentration, the pH of the solution needs to be controlled during all stages of the preparation process, so that the biopolymers are charged enough to avoid destabilisation processes [38].

In the literature, multi-layer emulsions with two [36], three [9] and up to four layers [40] of biopolymers have been produced. The properties of multi-layer emulsions strongly depend on the characteristics of the biopolymers used to assemble the different layers. For instance, Mun and co-authors reported that the particle size of a bi-layer emulsion (Tween 20-chitosan) was affected by the chitosan’s degree of deacetylation (DDA), presenting lower particle sizes than those emulsions containing the chitosan with the lowest DDA [41].

### 3.3. Liposomes

Liposomes are spherical structures consisting of polar lipids, which have hydrophilic and lipophilic groups within the same molecule [10]. Hydrophobic tails of lipids interact with each other, thereby forming bilayer structures that have an aqueous interior and at the same time are surrounded in aqueous media. Therefore, hydrophilic and lipophilic compounds can be simultaneously encapsulated in the aqueous core and the phospholipid bilayer, respectively [42].

According to the number of phospholipid bilayers contained within each structure and the size of the liposome, they are classified as small unilamellar vesicles (single layer and <0.1 μm), large unilamellar vesicles (single layer and 0.1–1 μm) and multilamellar liposomes (several layers and >1 μm) [43]. Different lipids have been employed for liposome formation, including phosphatidylcholine, sphingomyelin, phosphatidylserine, phosphatidylinositol and soybean lecithin. Methods to produce liposomes are based on the use of organic solvents, such as film hydration, ethanol injection and reverse-phase evaporation However, liposomes prepared with these techniques normally present large particle sizes and a broad size distribution. The incorporation of an additional step (extrusion, sonication or microfluidization) might be used as an efficient strategy to produce liposomes with small particle size and with high encapsulation capacity [44]. Solvent-free methods, such as pH-driven [45] and heating-hydrated components from liposomes in the presence of glycerol [46], are alternatives that can be used for liposome formation and further food application. However, this type of nanostructures is difficult to scale up due to the several steps needed for the preparation. Besides, liposome fabrication techniques have a considerable influence on the encapsulation efficiency of bioactive compounds. Dehydration/rehydration techniques have shown high efficiency in incorporating lipophilic bioactive compounds, such as retinol [47], while a significant decrease on encapsulation efficiency of resveratrol in liposomes from 97% to 44% was observed when they were prepared by the extrusion technique and sonication, respectively [48].

Recently, LbL techniques employed in emulsions have also been used for coating liposomes with different biopolymers, such as chitosan, pectin and carrageenan [49]. Another type of liposome is a niosome, which is formulated with non-ionic emulsifiers such as Span 40, Tween 20 or Tween 40. They are often chosen over liposomes because they are more stable and economic. However, there are only a few studies available on the formulation of niosomes containing lipophilic bioactive compounds [50].

## 4. Enhancing Functionality of Nanostructured Lipid-Based Delivery Systems

The successful delivery of encapsulated lipophilic bioactive compounds might be related to several factors, including the susceptibility of these nanostructures to undergo physical alteration when they are incorporated into foodstuffs or subjected to an in vitro gastrointestinal digestion. At the same time, effective nanostructures as delivery systems might be associated with their stability and digestibility, as well as bioaccessibility and absorption of health-related compounds. Thus, focusing research on studying all these processes is of great importance in order to cope with problems that might affect the overall functionality of nanostructures.

### 4.1. Enhancing Stability of Lipophilic Bioactive Compounds

Food processing and food matrix characteristics involve subjecting nanostructures to external environments that can negatively affect their integrity. To simulate these situations, nanostructures are normally exposed to different external or storing conditions (pH, ionic strength, light, temperature), followed by a monitorization of compound stability and/or physical stability of the system. Different studies about nanostructures under these conditions are described in Table 1.

On one hand, a high content of the lipophilic bioactive compound after exposure to external environments is important so as to maintain an overall functionality of the system. The principal advantage of nanostructured systems is their nanometric size, meaning that oil droplets have a large surface area. However, they are highly reactive with the surrounding media, and thus significant degradation of the encapsulated compound might occur. Teo and co-authors reported that the lutein degradation rate was strongly associated with the particle size of nanoemulsions, being higher as their particle size decreased from 147.3 nm to 68.8 nm [51]. Stabilisers also play a fundamental role in the stability of the encapsulated compound because they are deposited at the interface, thereby covering the oil droplets that have the lipophilic compound solubilised. It was reported a faster β-carotene degradation in nanoemulsions stabilised with Tween 20 than those with β-lactoglobulin [52]. Opposite results were observed by Young et al. [53], reporting a higher curcumin content in emulsions containing Tween 20 (>97.5%) compared to those containing whey protein (WPI) (<89.5%). The electrical charge of oil droplets, which is mainly attributed to the stabilisers employed to formulate nanostructures, might have a direct impact on the stability of the compounds. Negatively charged oil droplets (anionic particles) are easily reactive to metal ions present in the aqueous phase, leading to degradation processes of the encapsulated compounds.

Another way to retard encapsulated lipophilic compound degradation is to strengthen the physical barrier by covering the oil droplets with several stabiliser layers [35]. For instance, it was observed that adding a layer of chitosan to lecithin-covered oil particles containing citral (multilayer emulsions) was effective to retain a twofold amount of citral compared to the lecithin emulsions (single layer emulsions) after four weeks of storage (25 °C) [54]. Accordingly, other authors found higher retention of β-carotene in multilayer emulsions (WPI-gum arabic) than in single layer emulsions (WPI) at all the storage temperatures tested (25 °C, 37 °C and 45 °C) during 70 days of storage [55]. Similarly, applying a coating of pectin in the liposomes resulted in higher retention of resveratrol than in uncoated liposomes, avoiding oxidation and hydrolyzation of phospholipids processes and, therefore, leakage of encapsulated resveratrol [56]. Finally, the addition of water-soluble antioxidants, such as ascorbic acid, or oil-soluble antioxidants like coenzyme Q10, can delay the degradation of the encapsulated compounds in nanostructured systems by scavenging free radicals [57,58]. Some of the stabilisers employed in nanostructured systems production, including proteins and some biopolymers, have antioxidant capacity. Accordingly, it was suggested that the antioxidant capacity of guar gum used to formulate β-carotene-enriched liposomes contributed to avoid β-carotene degradation, leading to values of retention ≥88% after 95 days under refrigeration conditions [59].

On the other hand, instability processes occurring in nanostructures can be associated with a breakdown of the structure, resulting in a no longer encapsulation of lipophilic compounds. Consequently, unprotected compounds would be more susceptible to undergoing degradation processes. Therefore, the physical stability of nanostructured systems needs to be evaluated under different situations in order to understand their behaviour, so as to design stable systems. The nanometric size of nanostructured systems is associated with a reduction in the gravity force and Brownian motion, as well as with the prevention of destabilisation processes (flocculation, coalescence, sedimentation, creaming). Lee and co-authors observed that emulsions with a low particle size (≈66 nm) had better stability under different pH conditions (3–8), ionic strength (0–500 mM), thermal treatment (30–90 °C) and freezing/thawing (−4 °C, 48 h followed by 25 °C, 6 h) [60]. However, physical stability also lies on the composition of the nanostructures. Artiga-Artigas et al. [25] observed that the physical stability of curcumin-enriched nanoemulsions stabilised with lecithin strongly depended on the concentration of the emulsifier used, being those nanoemulsions containing concentrations over 1% the most stable emulsion. In the case of sucrose monopalmitate emulsions, they presented destabilisation processes (clarification, sedimentation, flocculation and coalescence) just hours after preparation. Because of the numerous layers deposited around the droplets, multi-layer emulsions might improve physical stability over a wide range of conditions. The physical stability of emulsions subjected to ionic strength changes (0–500 mM NaCl) was improved when they were covered with two (sodium dodecyl sulphate-chitosan) and three layers (sodium dodecyl sulphate-chitosan-pectin membranes) [61]. Another investigation concluded that the physical stability of multi-layer emulsions at different heat treatments highly depended on the biopolymer type employed for formulating these systems. Specifically, secondary emulsions containing lactoferrin-alginate were prone to droplet aggregation at temperatures over 60 °C, while droplet size remained unchanged up to temperatures of 90 °C when they contained lactoferrin-pectin (high and low methoxyl). Authors attributed these differences to the branches that pectin has, which would have provided both steric and electrostatic stabilisation [62].

**Table 1:** Recent studies about stability of nanostructured lipid-based delivery systems under different stressing conditions.

	Lipophilic Compound	Materials	Preparation Method	Stressing Conditions	Main Findings	References
Nanoemulsions	Curcumin	Lipid phase: corn oil Emulsifier: Tween 20, lecithin and sucrose monopalmitate	MF	Storage: 86 days at 25 °C	Lecithin-stabilised nanoemulsions were the most stable, while the rest undergone destabilisation processes after 24 h preparation.	[25]
	Ergocalciferol	Lipid phase: soybean oil Emulsifier: modified lecithin (ML), sodium caseinate (SC), decaglycerol monooleate (MO-7S)	HPH	pH conditions: 2–8 Ionic strength: 0–500 mM NaCl Thermal treatment: 80, 100 and 120 °C Dark storage 25 °C and 55 °C.	Physical stability depended on the emulsifier type. Stability of ergocalciferol in emulsion system decreased in order of ML>MO-7S≫SC during storage (55 °C for 30 days).	[63]
	Vitamin E	Lipid phase: SCT, MCT and LCT Emulsifier: Tween 80	LEM	Ionic strength: 0–500 mM NaCl pH: 2–8.5 Thermal treatment: 30–90 °C Storage: light/dark at 4, 25 and 40 °C	Nanoemulsions were physically stable at high temperature (≈90 °C), high ionic strength (≈500 mM) and long-term storage (60 days) under light and dark conditions (4–40 °C)	[64]
	β-carotene	Lipid phase: soybean oil Emulsifier: Ulva fasciata (UFP)	MF	Thermal treatment: 70,80, 90 and 100 °C pH: 3–7 α-tocopherol: 0–500 mg/kg EDTA: 0–500µL/L	β-carotene was highly sensitive to acidic conditions and extreme temperatures. Addition of EDTA or α-tocopherol increased the stability of β-carotene	[65]
Multi-layer emulsions	Astaxanthin	Lipid phase: flaxseed oil Coatings composition: Q-naturale-pectin-chitosan	LbL	Thermal treatment: 20, 30, 50 and 80 °C Ionic strength: 50–1000 mM NaCl pH: 2–8 Storage: quantification of astaxanthin for 15 days.	Multi-layer-coatings improved astaxanthin stability during storage, as well as physical stability at elevated ionic strengths and temperatures.	[66]
	β-carotene	Lipid phase: corn oil Coatings composition: lactoferrin-alginate-ε-poly-l-lysine	LbL	pH conditions: 2–11 Ionic strength: 0–500 mM NaCl Thermal treatment: 30–90 °C	β-carotene content decreased only 40% when emulsions were subjected at temperatures ≤70 °C, in acidic conditions and below 0.3 M NaCl.	[37]
Liposomes	Resveratrol	Lipid phase: phospholipid, cholesterol Coatings: low and high methoxy pectin	FD	pH conditions: 3, 5 and 7.4 Ionic strength: 0–200 mM NaCl Thermal treatment: 4 and 25 °C for 7 weeks	Low methoxy pectin improved physical stability of liposomes as well as resveratrol retention under different stress conditions.	[56]
	Curcumin	Lipid phase: phosphatidylcholine (98.1%) and lysophosphatidylcholine (0.7%) Modifier: Pluronic F127, F87 and P85	TFE + DHPM	pH conditions: 7.4, 8, 10 and 12 Thermal stability: 80 °C	Adding pluronics improved thermal and pH stability of liposomes.	[67]

SCT: short-chain triglycerides; MCT: medium-chain triglycerides; LCT: long-chain triglycerides; EDTA: ethylenediaminetetraacetic acid; DTA, MF: microfluidizer; HPH: high pressure homogenizer; LEM: low-energy method (emulsion phase inversion: catastrophic phase inversion); LbL: layer-by-layer technique; FD: film dispersion method; TFE: thin film evaporation; DHPM: dynamic high pressure microfluidization.

### 4.2. Enhancing Lipid Digestibility and In Vitro Bioaccessibility of Lipophilic Bioactive Compounds

Lipids present in nanostructured systems are important components because their hydrolysis will affect the release of encapsulated lipophilic compounds and, therefore, its bioaccessibility. Lipid digestion occurs mainly during the intestinal phase and consists of the hydrolysis of oil droplets, which produces free fatty acids, diacylglycerols and monoacylglycerols [68,69]. These products together with bile salts and other digestion components are responsible for producing mixed micelles, which incorporate and solubilise the released lipophilic bioactive compounds for further absorption [70,71]. Thus, comprehension about bioaccessibility of lipophilic bioactive compounds requires knowledge about the lipid digestion of the nanostructured systems.

Different factors related to nanostructures might influence on lipid digestibility and bioaccessibility, including their physicochemical properties and composition (Table 2). As a general rule, emulsions with an initial small particle size present a higher lipid digestibility and bioaccessibility compared with emulsions with larger particle sizes [72]. However, Lee et al. [60] observed that free fatty acids release was slightly higher in emulsions with a high particle size whereas no major differences in lipid digestibility between nanoemulsions (200 nm) and emulsions (10,000 nm) were found in another investigation [73]. These contradictory results might be related to the interfacial properties of the nanostructured systems, which in turn define their physicochemical properties. Furthermore, these properties might be associated with the susceptibility of nanostructures to undergo physical changes when they are subjected to an in vitro gastrointestinal tract (GIT). Specifically, nanostructured systems might be destabilised along the GIT because of the pH conditions during the different digestion stages, as well as the presence of salts and enzymes [74]. All these changes modify the initial properties of nanostructured systems and could probably affect lipid digestion [75] and, therefore, the bioaccessibility of the encapsulated compounds. In a recent study, it was observed that nanoemulsions stabilised with different emulsifiers (Tween 20, lecithin, sodium caseinate and sucrose palmitate) and concentrations (2%, 4% and 8%) behaved differently during lipid digestion, but a similar final lipid digestibility was obtained regardless of the composition of the nanoemulsions. However, the bioaccessibility results were strongly influenced by the emulsifier nature rather than by the lipid digestibility results, obtaining the higher β-carotene bioaccessibility in lecithin-stabilised nanoemulsions (23.5%) [76]. Similarly, it was reported that the highest vitamin E bioaccessibility was achieved when adding phospholipids to intestinal fluids [77]. Mixed micelles are composed of bile salts, phospholipids from intestinal fluids as well as lipid digestion products from enzyme action during the intestinal phase. The fact that phospholipids were present, either in nanoemulsions [76] or in intestinal fluids [77], would have contributed to the formation of mixed micelles and therefore increase their solubilisation capacity.

Nanostructures formulated with protein-based emulsifiers tend to present values of lipid digestibility similar [76] or even higher [78] compared to other emulsifiers, but poor bioaccessibility of the encapsulated compounds. Indeed, proteins are known to form complexes with some lipophilic compounds such as carotenoids, thereby promoting aggregation and precipitation of mixed micelles [79]. Poor digestibility has also been associated with the low stability of nanostructured systems along the GIT. If these systems became highly unstable and their particle size increase, the oil droplet surface exposed to enzymes would be lower, leading to low digestibility and bioaccessibility [75,80]. In this sense, biopolymers, such as mandarin fibre, modified starch or corn fibre (texturizing/gelling agents), can be added to emulsions in order to increase the stability of these systems under digestive conditions and, therefore, increase both the lipid digestibility and bioaccessibility of the encapsulated compounds [81,82,83]. An increase in lipid digestibility in protein-stabilised nanoemulsions containing dietary fibre has also been observed, thereby preventing droplet aggregation during in vitro GIT digestion, maintaining the integrity of the system, and thus ensuring lipase could easily access the oil droplets [84,85]. On the contrary, it was observed that the initial rate of lipid digestion and the final amount of free fatty acids (%FFA) release was reduced when emulsions contained pectin (0.5%). This behaviour was explained because biopolymers can interact with calcium ions, reducing diffusion processes essential for complete lipid digestion. Alternatively, biopolymers might increase the thickness of the interfacial layer covering the oil droplets, thereby preventing their hydrolysation [86].

Biopolymers, including pectin, alginate and chitosan, can be used to assemble the different layers that cover the oil droplets of nanostructures [87,88,89]. However, there is controversy about whether biopolymer layers have a negative or positive effect on the bioaccessibility of the encapsulated compounds. Some authors have observed no significant correlation between the number of layers and lipid digestibility [40,90]. Silva and co-workers reported a similar total release in free fatty acids after in vitro dynamic digestion for curcumin-loaded nanoemulsions (WPI) (96.14%) and multilayer nanoemulsions (WPI–chitosan) (95.52%) [36]. Nonetheless, a recent study reported that tertiary emulsions (lactoferrin/alginate/ε-poly-L-lysine) presented significantly higher β-carotene bioaccessibility (70.1%) compared to primary (30.2%) and secondary emulsions (35.26%) [37]. On the contrary, other authors have suggested that the deposition of extra layers at the droplets interface might decrease free fatty acids release during lipid digestion [89,91]. Less free fatty acids were released in secondary emulsions (lecithin–chitosan) compared with primary emulsions (chitosan) [92]. Accordingly, multi-layer emulsions formed with chitosan and soy protein isolate (SPI) presented a lower amount of free fatty acids (87.9%) than multi-layer emulsions prepared with alginate and SPI (104.1%) [93]. Therefore, using chitosan in nanostructured systems can negatively affect lipid digestion. Nonetheless, the deposition of a chitosan layer around liposomes (chitosomes) would have controlled the release of encapsulated carotenoids during GIT, favouring its bioaccessibility [94].

**Table 2:** Recent studies about in vitro lipid digestibility and bioaccessibility of encapsulated lipophilic bioactive compounds in nanostructured lipid-based delivery systems.

	Lipophilic Compound	Materials	Gastrointestinal Model	Main Findings	References
Nanoemulsions	β-carotene	Lipid phase: corn oil Emulsifier: Tween 20	Static in vitro gastrointestinal tract (GIT)	Lipid digestibility and β-carotene bioaccessibility increased from 34% up to 59% with decreasing particle size of emulsions.	[72]
	Curcumin	Lipid phase: corn oil Emulsifier: Tween 20, SDS or DTAB	Dynamic in vitro gastro-intestinal model (TIM)	Behaviour of nanoemulsions during in vitro digestion depended on the charge of the emulsifier	[95]
	Vitamin D3	Lipid phase: MCT, corn oil, fish oil, mineral oil, or orange oil Emulsifier: Q-Naturale	Static in vitro gastrointestinal tract (GIT)	Long chain triglycerides (corn oil and fish oil) were most effective at increasing vitamin bioaccessibility (≈80%).	[7]
	DHA algae oil	Lipid phase: DHA algae oil Emulsifier: Tween 40, sodium caseinate, soya lecithin	Static in vitro gastrointestinal tract (GIT)	Encapsulated DHA in nanoemulsions showed higher FFA release (40%–50%) compared to bulk DHA (≈20%).	[96]
Multilayer emulsions	Fish oil	Lipid phase: fish oil Layers assembly: Citrem–chitosan–alginate	Dynamic in vitro gastro-intestinal model (TIM)	Lipid digestion rate was decreased with multilayer coating	[91]
	Carotenoids	Lipid phase: MCT oil Layers assembly: soy protein isolate-alginate-chitosan	Static in vitro gastrointestinal tract (GIT)	Alginate coating had no effect on lipid digestibility (≈100%) and bioaccessibility of carotenoids (≈11%).	[93]
	Curcumin	Lipid phase: MCT oil Layers assembly: WPI–chitosan	Dynamic in vitro gastro-intestinal model (TIM)	The deposition of a chitosan layer did not affect lipid digestion (≈96%), but increased curcumin bioaccessibility (37.2%) compared to nanoemulsions (29.8%).	[36]
Liposomes	Curcumin	Lipid phase: cholesterol, phospholipid Modifiers: Pluronic F127, F87 and P85	Static in vitro gastrointestinal tract (GIT)	Curcumin loaded in pluronic-modified liposomes possessed increased bioaccessibility from 26.9% up to 43.3%.	[67]
	Curcumin	Lipid phase: phosphatidylcholine and α-phosphatidic acid Coating composition: chitosan	Static in vitro gastrointestinal tract (GIT)	Uncoated and coated liposomes presented similar results in curcumin bioaccessibility (≈70%).	[97]

Sodium dodecyl sulphate (SDS); dodecyltrimethylammonium bromide (DTAB); MCT: medium chain triglyceride oils; WPI: whey protein isolate; DHA: docosahexaenoic acid.

Other contributors or suppressor factors of lipid digestibility and bioaccessibility have been studied, including the chain length and degree of unsaturation of lipids used for nanostructure production. In general, no significant differences have been reported in the extent of lipid digestion for nanoemulsions containing long-chain triglycerides (LCT) or medium-chain triglycerides (MCT) [52,98]. Conversely, a higher rate and extent of lipid digestion as well as bioaccessibility was reported for LCT–vitamin E emulsions in comparison with MCT–vitamin E emulsions [99]. A recent study reported that vitamin D_3_-loaded nanoemulsions containing monounsaturated fatty acids (corn oil) were digested quicker than those oils containing polyunsaturated fatty acids (flaxseed and fish oil), which was explained by the conformation that the polyunsaturated fatty acids can adopt, thereby restricting enzyme action. Moreover, vitamin D_3_ bioaccessibility was higher in nanoemulsions formulated with corn oil (78%) than those formed with the rest of the oils (≈43%) [100]. Similarly, liposomes from phospholipids derived from milk fat globule membrane (MFGM) showed a lower free fatty acids release compared to those derived from soybean, which was explained because of the higher amount of saturated fatty acids that MFGM contained [101].

Finally, bioaccessibility of the encapsulated compounds can also be determined by the properties of the lipophilic compound. For instance, the intestinal release of different carotenoids loaded in liposomes changed depending on the type of carotenoid: lutein and β-carotene were released at a slower rate than lycopene and canthaxanthin [102]. This study concluded that polar carotenoids with a perpendicular orientation within the phospholipid bilayer of liposomes (i.e., lutein and β-carotene) would have favoured the carotenoid transfer to mixed micelles for further absorption.

### 4.3. Enhancing Absorption of Lipophilic Bioactive Compounds in Epithelial Cells

There are several lines of evidence to conclude that lipid-based nanostructures might be potential delivery systems for bioactive compounds. However, the efficacy of these bioactive compounds is directly associated with their bioavailability, which is the fraction of the bioactive compound that reaches the systemic circulation. Bioavailability of compounds involves several steps, including their release from the matrix, solubilisation within mixed micelles, permeability, absorption and secretion to the blood system [103]. A critical step of absorption is permeability (or cellular uptake), which can be defined as the lipophilic bioactive compound amount that enters within epithelial cells for later passage to bloodstream and target organs.

Before carrying absorption assays and after digestion processes, it is important to determine the cytotoxicity of the nanostructured systems so as not to compromise the survival of cells during tissue culture studies. Sadhukha and co-workers investigated the cytotoxicity of the digested nanoemulsions, which were composed by pellets, oil droplets and an aqueous fraction, the latter one being the most important part of the digesta since it has solubilised the lipophilic compound (micellar fraction). Surprisingly, the aqueous fraction was the most toxic to cells, which was explained by the free fatty acids generated during in vitro GIT digestion [104]. Besides that, other hydrophilic components from nanostructured systems, such as emulsifiers, have also been reported to affect the viability of cells [105,106]. Both studies analysed the cytotoxicity of several non-ionic emulsifiers in Caco-2 cells using the diphenyltetrazolium bromide test (MTT) and lactate dehydrogenase test (LDH). Polyethene glycols (Tweens and Labrasol) were more toxic in a dependent manner than polyethene glycols, being those with the highest HLB (Tween 20 and 60) the most toxic.

Many lines of evidence have demonstrated that the use of differentiated monolayers (Caco-2 and Caco-2/HT29-MTX cocultures) with tight junctions best represent the morpho-functional features of the intestinal barrier to model the absorption of lipophilic bioactive compound in epithelial cells [107]. This process consists of applying the digested sample at a suitable concentration in the apical compartment and incubate for at least 2 h. Finally, fractions from the apical (upper chamber), cell monolayer (in-between chamber) and basolateral compartment (lower chamber) are collected and analysed.

Numerous studies have investigated which properties of nanostructures might have a major influence on absorption of encapsulated compounds in tissue culture studies (Table 3). For instance, Andar et al. [108] observed a higher cellular uptake in Caco-2 cells for liposomes with small particle sizes (between 40 and 73 nm) compared to larger sizes (between 97 and 277 nm). Similarly, nanoemulsions exhibited a higher cellular uptake of lutein as their particle size diminished from 147.3 to 68.8 nm (147.3 nm) [51]. A great permeation through the cell monolayer as well as the large surface area would explain the high cell absorption in nanostructured delivery systems with low particle sizes.

Interfacial properties also have a strong influence on cellular uptake and absorption processes. In this sense, a recent study reported that the cellular uptake in Caco-2 cells of astaxanthin-loaded liposomes was improved when liposomes were formulated with 70% phosphatidylcholine [109]. On the other hand, another study described that cellular uptake of β-carotene in undifferentiated gastrointestinal epithelial cells (Caco-2 cells) depended on the emulsifier type added to the nanoemulsions rather than on the initial particle size of the nanoemulsions [110]. Investigations about different emulsifiers and combinations in oil-in-water emulsions concluded that they had a strong influence on the cellular uptake of carotenoids by the HT-29 cells. In this sense, oil-in-water emulsions formulated with a combination of whey protein (Biozate) and lecithin or sucrose laurate (L-1695) enhanced more than threefold the cellular uptake of lutein and astaxanthin, respectively [111,112]. Since the interfacial properties of the nanostructured systems govern the cellular uptake, a positive charge of the multi-layer emulsions containing chitosan would have increased the uptake efficiency compared to those containing WPI, which had a negative charge [36]. Hydrophilic polyelectrolytes, such as chitosan, can enhance the transport of these systems through Caco-2 cells via specific interaction between the nanostructured systems and the intestinal epithelium. Lipid composition is another parameter that might have a substantial influence on the permeability of lipophilic compounds. The impact of lipid phase-type (MCT, medium-chain triglycerides; and LCT, long-chain triglycerides) on bioavailability of vitamin E-encapsulated in quillaja saponin emulsions was investigated and it was concluded that LCT emulsions presented higher bioavailability (11.7%) than MCT emulsions (4.3%) [113]. Opposite results were reported by Liu and co-authors, presenting a higher permeability coefficient of pterostilbene across the cell monolayer in those nanoemulsions containing MCT (8.21 × 10^−6^ cm s ^−1^) compared to those with LCT (≈5.10 × 10^−6^ cm s^−1^) [114].

Co-culturing Caco-2 cells with HT-29MTX adds a further layer of mucus complexity to more closely resemble the in vivo environment [115], but reduces permeability rates, which may hamper compound detection. Accordingly, a lower permeability of β-carotene from liposomes was reported using an in vitro, 21 days differentiated co-culture model with Caco-2/HT29-MTX compared to 21 days differentiated Caco-2 cells [116]. Interaction with the mucus produced by the Caco-2/HT29-MTX cell line reduces the permeability of mucoadhesive lipophilic molecules or large molecules due to steric blocking [117]. Detection of the target compound after absorption experiments by determining their bioactivity using other cells is an alternative that it has been used by Sabouri et al. [118].

**Table 3:** Recent in vitro studies about different nanostructured lipid-based delivery systems encapsulating lipophilic bioactive compounds in epithelial cells.

	Lipophilic Compound	Composition	In Vitro GIT Digestion	Epithelial Cells	Main Findings	References
Nanoemulsions	Conjugated linoleic acid (CLA)	Lipid phase: Soybean oil (14% *v*/*v*) Emulsifier: Soy protein isolate (4% *v*/*v*)	Yes	Differentiated Caco-2 cells	No significant differences on CLA bioavailability for all emulsion treatments	[119]
	Curcumin	Lipid phase: soy oil (40% *v*/*v*) Emulsifier: Tween 20 (4% *v*/*v*) or Poloxamer 407 (4% *v*/*v*)	No	Differentiated Caco-2 cells	Curcumin uptake was significantly affected by the type of interface, being higher when emulsions were stabilised with Poloxamer 407.	[120]
	β-carotene	Lipid phase: sunflower oil (10% *w*/*w*) Emulsifier (1% *w*/*w*): whey protein isolate (WPI), sodium caseinate (SCN) and Tween 80 (TW80).	Yes	Undifferentiated Caco-2 cells	Sodium caseinate-stabilised emulsion showed the highest cellular uptake of β-carotene, followed by TW80- and WPI-stabilised emulsions.	[110]
	Vitamin D	Lipid phase: canola oil (0.5, 1, 2.5, 5% *w*/*v*) Emulsifier: pea protein (1, 5, 10% *w*/*v*)	No	Differentiated Caco-2 cells	Cellular uptake was higher for small sized nanoemulsions (233 nm) and protein-based-nanoemulsions.	[121]
Multilayer emulsions	Curcumin	Lipid phase: medium chain triglycerides (MCT) (10% *w*/*w*) Emulsifier: whey protein isolate (WPI) (1.5% *w*/*w*) Coating composition: chitosan	No	differentiated Caco-2 cells	Chitosan layer significantly increased the apparent permeability coefficient of curcumin through Caco-2 cells by 1.55-folds.	[36]
Liposomes	Epigallocatechin-3-gallate	Lipid phase: soybean oil (7%) Emulsifier: sodium caseinate (0.35%) Stabiliser: high methoxyl pectin (0, 0.45%)	No	Differentiated Caco-2 cells and coculture Caco-2/HT29-MTX	Mucus layer was associated with a lower recovery of Epigallocatechin-3-gallate after uptake experiments	[116]
	Curcumin	Lipid phase: phospholipid, cholesterol Emulsifier: Tween 80	No	Undifferentiated Caco-2 cells	Curcumin loaded in nanoliposomes exhibited lower curcumin cellular uptake than free curcumin	[122]
	Astaxanthin	Lipid phase: soybean with phosphatidylcholine (PC)	No	Differentiated Caco-2 cells	Cellular uptake of astaxanthin-loaded liposomes containing 70% PC was significantly higher than that of 23% PC-containing liposomes	[109]

## 5. Application of Nanostructured Lipid-Based Delivery Systems

Functional foods are defined as food products that have been fortified or enriched with compounds that provide health benefits. Because of the promising properties that nanostructured systems offer, they can be applied in several foods either in a dry/solid or liquid form to produce functional foods.

Nanostructured systems can be transformed into a dehydrated or solid state by spray-drying or freeze-drying techniques. Both processes consist of drying the product so as to obtain a powder with the encapsulated and entrapped compound of interest. In spray drying, water is evaporated by using hot air, while in freeze drying samples are frozen and then removing the liquid by sublimation under a vacuum [123]. One study obtained a lycopene powder through spray drying for further application within cakes. As a result, a pigmented cake was obtained, indicating that lycopene was released from the powder during cake preparation and coloured the dough homogenously [124]. Other authors have found evidence on using freeze drying to encapsulate bioactive compounds for an efficient food functionality. In this sense, yogurt fortified with freeze-dried capsules containing carotenoids and polyphenols exhibited a high retention of both bioactive. Furthermore, good acceptability scores in terms of colour and appearance were obtained for fortified yogurts [125].

Alternatively, nanostructured systems can be incorporated into foodstuffs either as such (beverages) or as edible coatings (fruits and vegetables). On one hand, Marsanasco et al. [126] incorporated both vitamin E- and vitamin C-enriched liposomes to an orange juice and observed that the antioxidant activity of both vitamins was protected before and after pasteurisation treatments. Furthermore, orange juice organoleptic properties were not altered, and its microbial stability was improved for over a month. On the other hand, edible coatings consist of a thin layer of nanostructured emulsion systems that are applied to foods by different techniques, such as spraying, dipping and electrospraying. This layer can act as a physical barrier and block oxygen, moisture and solute movement, without affecting the properties of the food [127]. For instance, multi-layered emulsions have been used as edible coatings to provide a better quality and visual appearance to strawberries [128]. Nanoemulsions containing eugenol and *Aloe vera* were also applied to shrimps, resulting in a quality improvement of this food product (drip loss, colour changes, textural disintegration and deterioration was reduced) as well as in a retardation of oxidative reactions during seven days of storage [129]. In addition, nanostructured systems are a feasible approach to extend the shelf life of food as well as add extra nutritional value. The microbial stability of low-fat cheese was improved, and additional nutritional characteristics were provided to the food product by applying a nanoemulsion-based edible coating, carrying oregano essential oil and mandarin fibre [26].

## 6. Concluding Remarks and Future Prospects

Bioactive compounds are health-related compounds of interest not only because they are involved in biological processes but also for their specific functions for health and well-being. It is widely recognized that nanostructured lipid-based delivery systems are a feasible option to encapsulate and improve the functionality of such lipophilic bioactive compounds. At the same time, the successful delivery of bioactive compounds might be associated with the stability and digestibility of nanostructured systems, as well as bioaccessibility and absorption. Different characteristics from nanostructured lipid-based delivery systems can either strengthen or reduce their functionality, including interfacial properties of and/or carrier oil-type, among others.

However, most of the in vitro models used for studying delivery of encapsulated compounds within nanostructured systems do not include the large intestine step, where interaction with microbiota occurs. The need for accurate in vitro methods to study digestion and fermentation processes has become increasingly important given the recently recognised role of the large intestine in vital functions, nutrition and gut health. In addition, most of these nanostructures have only been developed at the scale laboratory level, which limits its extrapolation to in vivo studies. Further in vivo studies (animal tests) are needed not only to validate the results obtained from in vitro experiments but also to obtain information prior to embarking on intervention trials with human subjects.

Another latent concern is the lack of information about possible interactions between nanostructured lipid-based delivery systems and other food components, as well as their accumulation, toxicity and long-term consumption. Even though nanostructured delivery systems have been associated with the quality, safety and functionality enhancement of encapsulated bioactive compounds, there is a global concern regarding both the toxicological and regulation aspects when these systems are applied to foodstuffs and then consumed. Now that the food and beverage industry is continuously developing novel food products in which nanotechnology is involved, the implication of authorities is essential in order to establish a law when using nanotechnology along the food chain for future food applications.
